# Complications after Dynamic Hip Screw Osteosynthesis of Proximal Femoral Fractures at Army Instructional Hospital-Libreville

**DOI:** 10.1155/2021/4177203

**Published:** 2021-10-04

**Authors:** F. M. Bombah, M. Diawara, B. Y. Ekani, T. Nana, A. Mikiela

**Affiliations:** ^1^Department of Surgery, Faculty of Medicine and Pharmaceutical Sciences, University of Douala, Cameroon; ^2^Orthopedic and Traumatologic Surgery Department, Army Instructional Hospital, Libreville, Gabon; ^3^Department of Surgery, Faculty of Medicine and Pharmaceutical Sciences, University of Buea, Cameroon

## Abstract

The DHS system is an effective means of open reduction and internal fixation of proximal femur fractures. Postoperative complications are little described and deserve to be studied for better preventive and curative treatment. We report the observations of five (5) patients who presented postoperative complications specific to the DHS system at army instructional Hospital-Libreville. These described complications are related to deterioration of internal fixation leading to callus, nonunion, or infection. Those found are the cut out phenomenon, avascular necrosis of the femoral head, and the fracture of the DHS system. All these complications required surgical revision without functional repercussions at the last follow-up. Complications of internal fixation by the DHS system can be avoided by rigorous asepsis, adequate indications for surgery, and rigorous surgical techniques. Good management can restore hip function.

## 1. Introduction

The DHS system is an effective means of open reduction and internal fixation of proximal femur fractures [1]. It was developed from 1980 by Richards Manufacturing and has been the subject of numerous studies [2, 3]. It has very precise indications and requires practical experience. Postoperative complications are poorly described [4] and deserve to be studied for better preventive and curative treatment. We report the observations of five (5) patients who presented postoperative complications specific to the DHS system.

## 2. Observations: (Table 1)

### 2.1. Case 1

**Figure 1:**
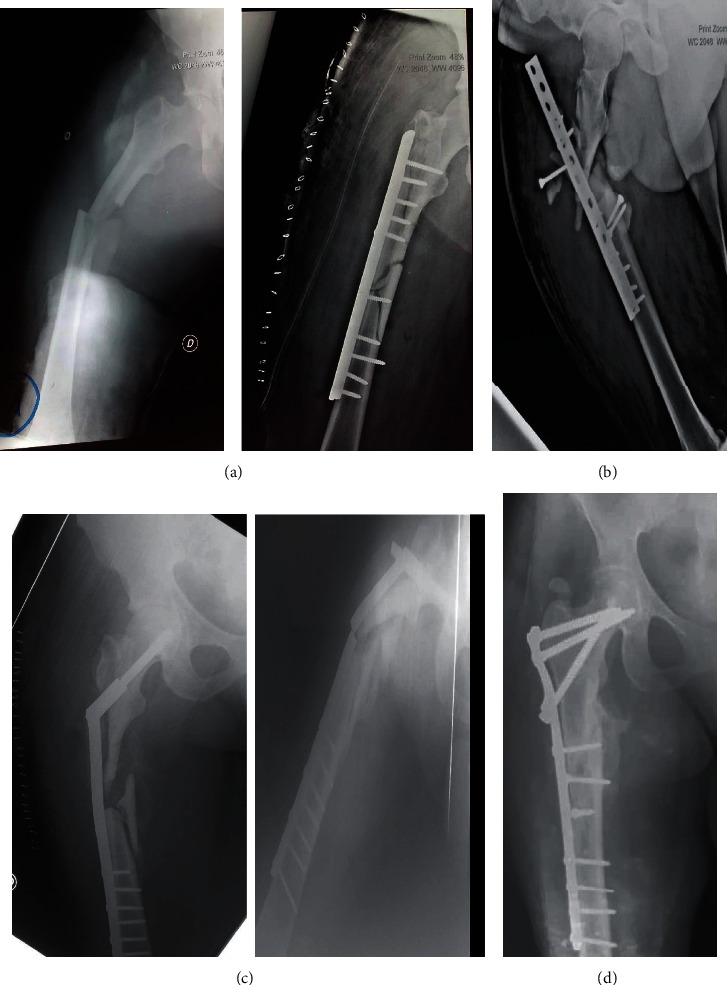
X-ray of case 1: ((a) subtrochanteric fracture; (b) missed fixation of DCP; (c) atrophic nonunion with fracture of the DHS system; (d) locked plate osteosynthesis).

42-year-old patient, motorcycle driver, was a victim of a blunt trauma to the right thigh after a road traffic accident on his motorcycle. The diagnosis of subtrochanteric fracture classified 32C3 according to AO/OTA (Figure 1(a)) is made and a dynamic compressive plate osteosynthesis performed in a clinic. The patient is referred to us 2 weeks postoperatively for posttraumatic plate removal (Figure 1(b)). The plate was deposited, and an osteosynthesis by the DHS system was performed. The evolution after six (6) postoperative months is marked by atrophic pseudarthrosis and a rupture of the plate in the 8th month (Figure 1(c)). The management consisted of a cure of nonunion, a spongy graft, and the internal fixation by a locking plate for proximal femur (Figure 1(d)). At the last follow-up (9 months), the functional [5] and radiological results (presence of callus) were very good.

### 2.2. Case 2

**Figure 2:**
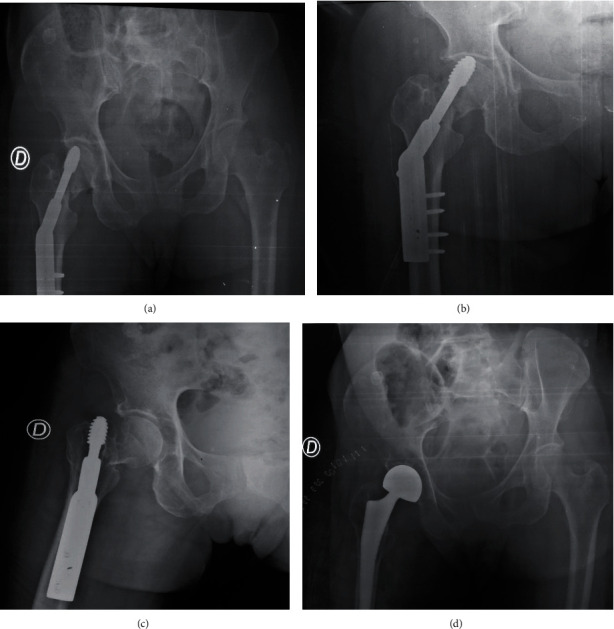
X-rays of patient 2. (a) DHS osteosynthesis of neck fracture. (b) Neck pseudarthrosis with phenomenon. (c) Cut out phenomenon with cephalic osteonecrosis. (d) Right hemiarthroplasty.

A 64-year-old trader was brought in an hour after trauma to the right hip in a car accident. The clinical and paraclinical examination made it possible to make the diagnosis of transcervical fracture of the femoral neck classified 31B2.3 according to AO/OTA (Figure 2(a)). The DHS was applied 5 days after the trauma under image intensifier. The course is marked by pain in the hip, pseudarthrosis of the fracture site, and necrosis of the femoral head and a gradual migration of the DHS system objectified in the 4th (Figure 2(b)) and 6th month (Figure 2(c)) postoperatively. The management consisted of a hip hemiarthroplasty (Figure 2(d)). At the last follow-up (18 months), the functional result was very good according to the PMA scale [5].

### 2.3. Case 3

**Figure 3:**
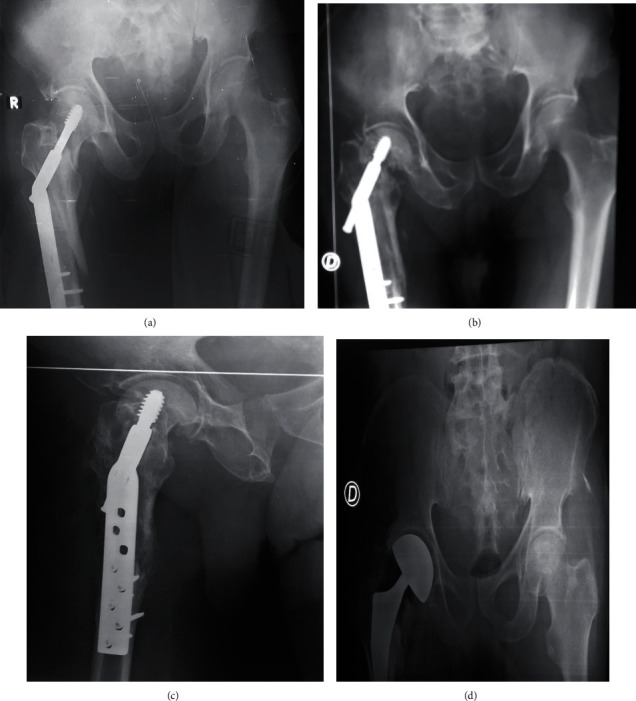
X-rays of patient 3. (a) Osteosynthesis y DHS. (b) Subtrochanteric malunion with pseudarthrosis of femur neck. (c) Cut out phenomenon. (d) Hemiarthroplasty.

A 56-year-old female farmer was received one hour from trauma to her right hip after falling from a motorcycle. The diagnosis of a basicervical fracture classified 32A2 according to AO/OTA (Figure 3(a)). A 7-hole DHS was installed. The evolution is marked by a vicious callus in the subtrochanteric region in the 4th postoperative month and pseudarthrosis of the femoral neck (Figure 3(b)). In the 6th postoperative month, osteonecrosis of the femoral head and dynamic screw migration is observed (Figure 3(c)). The DHS was removed, and an uncemented total hip prosthesis was placed. At the last follow-up (24 months), the functional and radiological results were satisfactory according to the PMA scale [5].

### 2.4. Case 4

**Figure 4:**
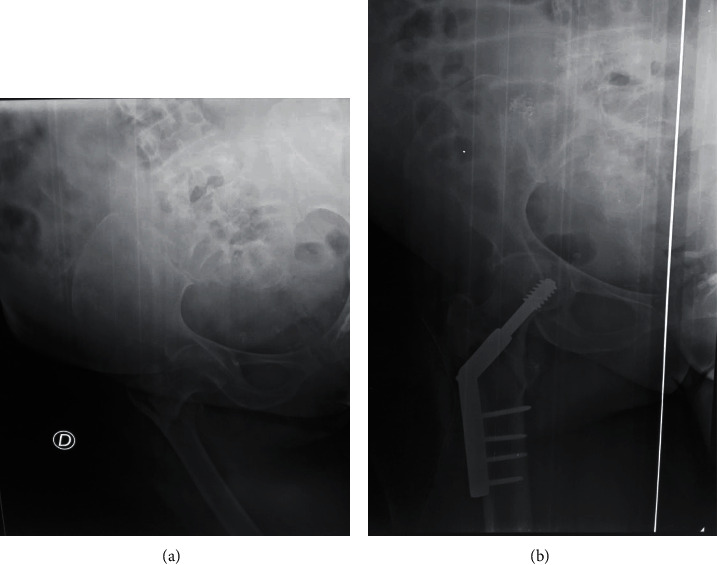
X-rays of patient 4. (a) Pertrochanteric fracture type 31A1.3 AO/OTA. (b) Osteosynthesis by DHS.

A 65-year-old male farmer sustained injuries when a motorbike hit him onto his right hip. At presentation, he was hemodynamically stable, and radiographs confirmed a minimally displaced pertrochanteric fracture of the right hip class 31A1.3 according to AO/OTA (Figure 4). The fracture was stabilized using DHS system after 72 h. The patient had an uneventful peri-operative period and was allowed partial weight bearing. At his six week outpatients' clinic appointment, radiographs showed callus and sliding screw fracture. He was therefore permitted full weight bearing after the removed of DHS system. One year after surgery, radiographs showed vicious callus with very good functional result [5]. He has returned to full-time work and is able to participate in low-impact recreational sporting activity.

### 2.5. Case 5

**Figure 5:**
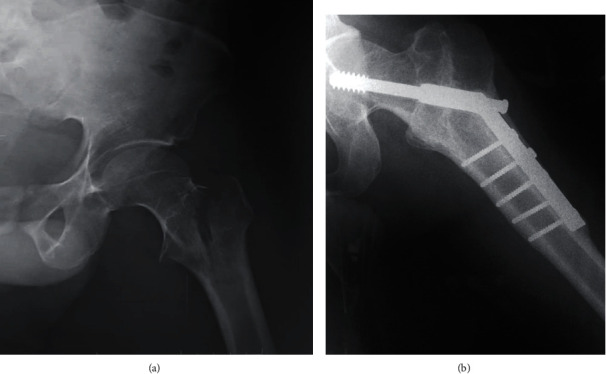
X-rays of patient 5. (a) Pertrochanteric fracture of the left hip class 31A1.2 according to AO/OTA. (b) Osteosynthesis by DHS.

A 46-year-old male driver was involved in a high-speed motorbike accident in which he sustained injuries onto his right hip. At presentation, he was hemodynamically stable, and radiographs confirmed a minimally displaced pertrochanteric fracture of the left hip class 31A1.2 according to AO/OTA (Figure 5). The fracture was stabilized using the DHS system (Figure 4(b)) after 3 days. The patient had an uneventful peri-operative period and was allowed partial weight bearing. At his nine month outpatients' clinic appointment, suppuration next to the operative scar is the objective, and radiographs showed callus at the proximal femoral fracture. Staphylococcus aureus has been identified. The DHS system was removed followed by necrosectomy, washing, and drainage. He has returned to full-time work and is able to participate in recreational sporting activity.

## 3. Discussion

Postoperative complications of DHS are rare and not enough described [4]. The various complications described are linked to deterioration of internal fixation leading to vicious callus, nonunion, or infection [1]. These complications are the cut out phenomenon, avascular necrosis of the femoral head, fracture of the DHS system, femoral plate fracture, and progression of coxarthrosis [4]. They represent a rate of 6% (22/367) according to Hrubina et al. [4]. We found 5 (6.5%) cases out of 77 internal fixation by DHS. We will discuss the indications, complications, and end treatments of our patients in light of the literature.

The DHS system is indicated mainly for trochanteric fractures. It is easy to set up and gives good results. However, it has limitations in cases of fracture of the lateral pillar of the upper extremity of the femur and comminution of the greater trochanter (case 3) [1].

Infection, although late, is a rare and serious complication. It can involve the vital and functional prognosis of the patient. We find 1 case of infection with Staphylococcus aureus. It is comparable with other relevant studies [6].

Malunion is a frequent complication of trochanteric osteosynthesis 12% for Decoulx and Lavarde [7] and lavender and 5% for Hrubina et al. [8]. It is due to an overestimation of bone quality and/or a technical error. We found 3/77 (3.9%) vicious calluses, one in the trochantero-diaphyseal region (patient 3) and 2 on the trochanteric mass in patient 4 and patient 5. These vicious calluses were tolerable and were not need a secondary realignment.

Pseudarthrosis is rare in the trochanteric mass because the vascularization is excellent [1]. On the femoral neck, there is a risk of cervical nonunion, whether or not associated with cephalic necrosis [9]. We noted 2 cases of nonunion of the cervix with avascular necrosis of the femoral head in patient 2 and patient 4. Hrubina et al. found 2 (0.5%) cases of nonunion in a series of 367 osteosynthesis by the DHS system.

Cut out phenomenon is a complication described in cases of osteoporosis in the elderly [1, 4]. It is also described in cases of targeting errors or progressive impaction of the fracture site. We find 3 cases (case 2, case 3, and case 4) in elderly patients with progressive impaction of the fracture site [10].

There are few articles about the metal breakage after the DHS osteosynthesis. For Hrubina et al. [8], there is metal breakage of K-wire, cortical screws, and sliding screw. We have seen a metal breakage of plate (case 1) and sliding screw (case 4).

## 4. Conclusion

Postoperative complications from internal fixation by DHS are at army instructional Hospital in Libreville. They are sometimes result from the failings of surgeons. They can be avoided by rigorous asepsis, adequate surgical indications, and rigorous surgical techniques. Good management of its complications can restore hip function.

## Figures and Tables

**Table 1:** Case series.

Cases	Age	Sex	Diagnostic	AO/OTA	Complication			Treatment
					Disturbed healing	Mechanical	Vascular	
1	42	M	Subtroch	32B2a	Subtrochanteric pseudarthrosis	DHS plate fracture	/	Plate
2	64	M	Transcervical	31B2.3	Neck pseudarthrosis	Cut out phenomenon	Cephalic avascular necrosis	PHRA
3	56	F	Pertroch.	32A2	Neck pseudarthrosis	Cut out phenomenon	Cephalic avascular necrosis	THRA
4	65	M	Pertroch.	31A1.3	Malunion	Cut out phenomenon, DHS sliding screw fracture	/	Remove of DHS system
5	46	M	Pertroch.	31A1.2	Infection	/	/	Necrosectomy, washing drainage
